# Multiple dentigerous cysts in a patient showing features of Gorlin-Goltz syndrome: A case report

**DOI:** 10.1016/j.ijscr.2023.109156

**Published:** 2023-12-13

**Authors:** Mohammad H Al-Shayyab, Ra'ed Hisham Aldweik, Mohammad Alzyoud, Aya Qteish

**Affiliations:** a Department of Oral and Maxillofacial Surgery, Oral Medicine and Periodontology, School of Dentistry, The University of Jordan, Amman 11942, Jordan; b Department of Pathology, School of Medicine, the University of Jordan, Amman 11942, Jordan

**Keywords:** Gorlin-Goltz syndrome, Dentigerous cyst, Third molar, Case report

## Abstract

**Introduction and importance:**

The association between Dentigerous cysts (DCs) and Gorlin-Goltz syndrome (GGS) was claimed theoretically in a very few reports, with very few clinical foundations. The aim of this report was to present a unique case of multiple DCs in the mandible in a patient showing features of GGS.

**Case presentation:**

A 63-year-old male patient presented with multiple cyst-like lesions in the mandible associated with some clinical and radiological features of GGS, and that raised the suspension of odontogenic keratocyst (OKC). The patient underwent marsupialization and enucleation of these cysts, and the histopathological examination confirmed the diagnosis of DCs.

**Clinical discussion:**

In this report, the patient presented with symptoms related to multiple unilocular radiolucent lesions found in the mandible and the clinical and radiological features were highly suggestive of OKCs associated with GGS. However, the perioperative findings raised the suspicion of DCs, which was confirmed by histopathology. Interestingly, GGS is an inherited autosomal dominant disorder arising from mutations in the patched tumor suppressor gene (PTCH). Previous studies showed this gene alteration in DCs; this can possibly be implicated in the pathogenesis of the association found in this report.

**Conclusion:**

This report presented a case of bilateral DC in the mandible in a patient showing features of GGS. Therefore, this report verified the very rare association between DC and GGS. This may help dentists and physicians in reaching an accurate and early diagnosis of GGS.

## Highlights

• Multiple jaw cysts can raise the suspicion of Gorlin-Goltz syndrome.
• Dentigerous cysts are considered as minor criteria for diagnosis of Gorlin-Goltz syndrome.
• PTCH gene mutation may be implicated in the association between Gorlin-Goltz syndrome and dentigerous cysts.

## 1 Introduction

Gorlin-Goltz syndrome (GGS) is a rare multisystem disorder first described by Gorlin and Goltz in 1960. Its incidence is estimated to be around 1 in 50,000 to 1 in 150,000 in the general population, with regional variations. The current diagnostic criteria for GGS involve a combination of major and minor criteria [1,2]. Major criteria contain the prevalent lesions such as multiple basal cell carcinomas, OKCs, and skeletal anomalies. On the other hand, minor criteria include less frequent manifestations like bone cysts in the hands, vertebral alterations and macrocephaly [3].

The DC appears radiographically as a well-defined unilocular radiolucency, with an impacted tooth that sometimes resembles the radiological features of OKC [4,5]. Clinical reports verified the possible association between this cyst and number of syndromes including cleidocranial dysplasia and Maroteaux-Lamy syndrome [6].

The association between DCs and GGS has been claimed in scanty literature [7]. To the best of the authors' knowledge, this association was reported in few published clinical cases [3,[8], [9], [10]]. Therefore, the aim of this report was to present a unique case of multiple DCs observed in a patient showing features of GGS. This presentation may help in verification of this association.

The work of this report has been reported in line with the SCARE criteria [11].

## 2 Case presentation

**Fig. 1:**
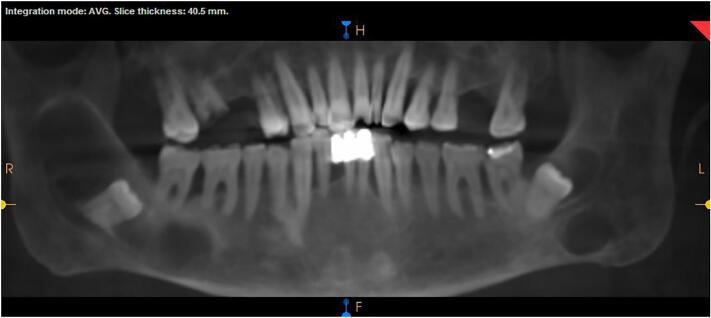
Reconstructed orthopantomograph from the patient's Cone Beam Computed Tomography showing three cyst-like lesions.

A 63-year-old male patient with history of hypertension, hyperthyroidism and radioactive iodine exposure, presented to the Emergency Department at JUH in September 2022 as a case of right buccal swelling associated with pain and limitation of mouth opening with two-day duration. Orthopantomograph (OPG) and computed tomography (CT) were ordered and showed three cyst-like lesions. In addition, the CT scan also showed adjacent inflammatory changes and the diagnosis was right buccal space infection related to an infected cystic lesion. The patient was vitally stable and discharged home on oral antibiotics and analgesia. One week later, a cone beam computed tomography (CBCT) were taken to further evaluate the three lesions: the larger one was in the right side and showed unilocular radiolucency measuring 6 × 1 × 2.5 cm, and was associated with the impacted right third molar; the left side contained two lesions, one above the ID canal measuring 1.5 × 0.7 × 1 cm and was associated with the lower left wisdom tooth, and the other one was below the ID canal measuring 1.5 × 0.5 × 1.6 cm and diagnosed as Stafne's bone cyst (SBC), as it was asymptomatic and appeared to housing an accessory part of the submandibular gland (Fig. 1).

**Fig. 2:**
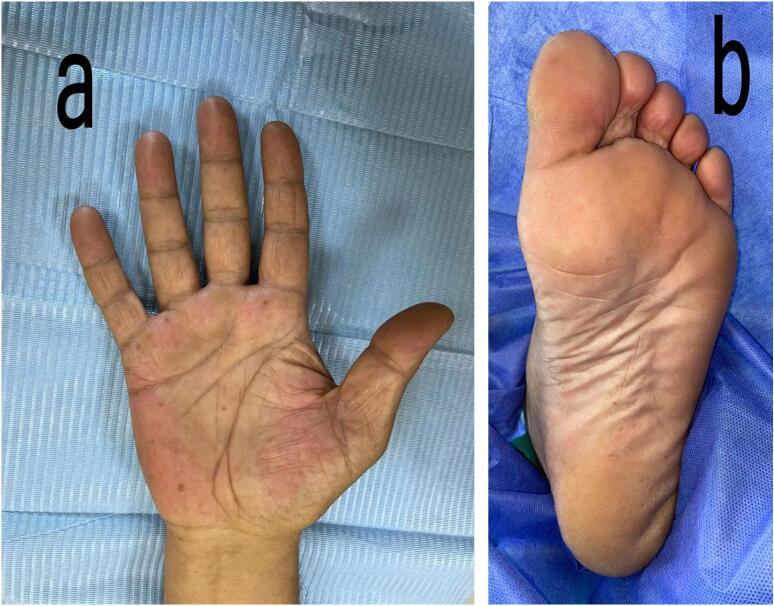
a Multiple palmer pits. b Multiple planter pits.

**Fig. 3:**
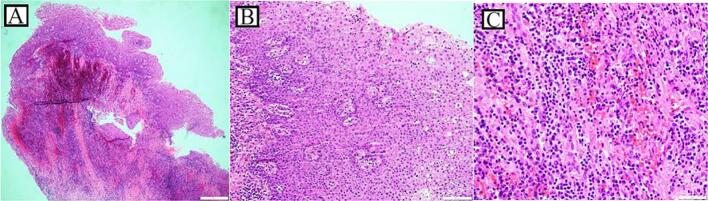
(A and B): Cyst wall lined by hyperplastic squamous epithelium, H&E, (4× and 20×), (C): The cyst wall is infiltrated by neutrophils, lymphocytes and plasma cells, H&E, (40×).

The multiple cyst-like lesions on the jaw raised the suspicion of GGS, so that a thorough clinical and radiological examination done; in addition to the CT scan and the CBCT, a chest X-ray reported no bifid ribs or calcified falx cerebri. However, multiple facial dermal calcinoses were noticed on the CT scan. In addition, some clinical features were noticed like palmer and planter pits (Fig. 2a, b), and multiple benign dermal cysts, with no features or history of basal cell carcinoma (BCC). Therefore, GGS was then considered and OKC was the provisional diagnosis for the lesions located above the ID canals, which were planned for management under general anesthesia (GA). Perioperatively, aspiration of both cysts showed a straw colored fluid, the left cyst underwent enucleation and extraction of the involved third molar, and the right one underwent incisional biopsy and extraction of the involved third molar tooth and marsupialization by suturing the cystic lining to the oral mucosa. A decompression tube was then inserted and fixed to the adjacent molar tooth. Unexpectingly, the histopathological examination of the specimens of both lesions showed thick lining and revealed multiple fragments of fibrous connective tissue lined by hyperplastic none-keratinized squamous epithelium with focal neutrophilic infiltration. The cyst wall was infiltrated by mixed inflammatory cells composed of neutrophils, lymphocytes and plasma cells, with rare microcalcifications seen in the left cyst. Occasional mucous cells were seen in the lining epithelium of the left cyst. No parakeratosis or keratin flakes were found in both cysts (Fig. 3). Therefore, a definitive diagnosis of DC was confirmed.

**Fig. 4:**
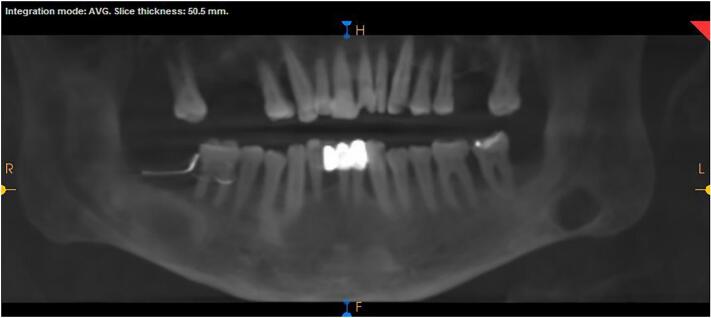
Follow up radiograph after 10 months showing decreasing in the cystic size with bony filling and the decompression tube.

**Fig. 5:**
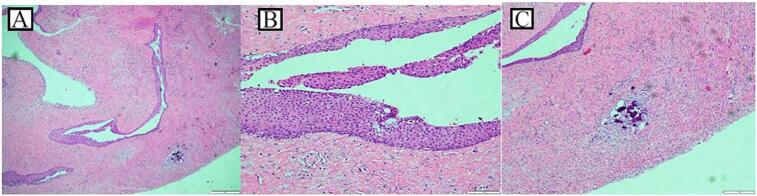
(A and B): Cyst wall lined by stratified squamous epithelium with occasional mucous cells, H&E, (4× and 20×). (C): Microcalcification, H&E, (10×).

Eleven months later, the OPG and CBCT showed good bony filling of the lesions with small remaining radiolucency measuring 4.5 × 0.7 × 1.1 cm (Fig. 4) in the marsupialized lesion, which was then enucleated and the histopathology report again confirmed the diagnosis of DC (Fig. 5).

## 3 Discussion

GGS is an inherited autosomal dominant disorder. This condition exhibits high penetrance and varying levels of phenotypic expressivity. In 1960, Gorlin and Goltz initially described the syndrome by three primary characteristics: the presence of multiple BCCs, OKCs, and skeletal abnormalities [3].

Unilocular radiolucency in the mandible includes many differential diagnoses like OKC, DC, ameloblastoma, simple bone cyst, giant cell granuloma and others [12]. However, the well-defined non-corticated margins, the location, the uniform and multiple presentation of the lesion, and the nature of the internal bony septa were all suggestive of a cystic nature, most commonly OKC and DC, of the lesion presented herein instead of the tumors or the simple bone cyst included in the differential diagnosis [12]. OKCs are benign jaw tumors and tend to occur as unilocular radiolucent lesions when small, while larger lesions tend to be multilocular extended mesiodistally with minimal mediolateral bone expansion. Their lining is thin stratified parakeratinized epithelium and usually associated with an impacted tooth. On aspiration, a creamy cheese-like material and keratin or keratin-like smell are commonly observed. Multiple lesions occurring in more than one jaw quadrant are highly suggestive of GGS [12,13]. In contrast, DCs occur due to the accumulation of fluid between the crown of an unerupted tooth and reduced enamel epithelium [14]. On radiographs, they appear as unilocular well-defined radiolucent lesions around an unerupted tooth crown, not characteristically extended or expanded in any direction [12]. The lining of the cyst is thick non-keratinized stratified squamous epithelium resembling reduced enamel epithelium, with a fibrous wall surrounding the lining from outer side with an inflammatory infiltrate in this fibrous area. On aspiration, straw-colored fluid is commonly observed [15]. In this report, the patient presented with symptoms related to multiple unilocular radiolucent lesions and the clinical and radiological features, particularly the mesiodistal extension of the right cystic lesion, were highly suggestive of OKCs commonly associated with GGS. However, the perioperative findings of thick lining and the straw colored aspirate raised the suspicion of DCs, which was confirmed by histopathology. Therefore, the similar clinical and radiological features of both OKC and DC sometimes make the final diagnosis to base on the histopathological features [16].

Bilateral and multiple DCs have been claimed theoretically to occur in association with GGS in the literature [7]. However, only four published case reports regarding this association were identified in the English literature: the first was published by Cawson et al. [8], in 1964, who presented three cases of GGS associated with DCs. However, the authors stated that cysts were apparently DCs based on confusing histopathological features with OKC; the second was published by Nakajima et al. [9], in 1977, but the authors used the term ‘dentigerous keratocyst’, which was never used before and thereby, was misleading; the third report was published by Anehosur et al. [10], in 2009, in which the patient diagnosed with multiple cysts including DCs and OKCs; the fourth report was published by Senayr et al. [3], in 2021, in which the authors stated that the lesion initially diagnosed in their report as an infected DC was possibly OKC. Therefore, this report presented a unique case that verified the very rare association between GGS and multiple DCs. Interestingly, a clinical diagnosis of GGS can be established when there are either two major criteria or one major and two minor criteria [3]. DCs were suggested as part of the minor criteria for diagnosis of GGS [3]. In this report, the patient had palmer and planter pitting (major), dermal calcinosis (minor) and multiple DCs (minor), so that the patient was diagnosed by having one major and two minor criteria.

Recently, the gene for GGS was cloned and shown to be the human homologue of the Drosophila segment polarity gene Patched (PTCH), a tumor suppressor gene. The PTCH gene encodes a transmembrane protein that acts in opposition to the Hedgehog signaling protein, controlling cell fates, patterning, and growth in numerous tissues, including teeth. This mutation that can affect the developing dental tissues may explain the mechanism of occurrence of OKC in association with this syndrome [17]. Other studies [18] also showed PTCH gene alteration in DCs and that can possibly explain the association between these cysts and the GGS found in this report.

## 4 Conclusion

This report presented a unique case of bilateral DCs and a SBC in the mandible in a patient showing features of GGS. Cystic lesions in GGS need to be diagnosed by assessing their radiographic appearances, the contents obtained on aspiration, and by evaluating the consistency of the cystic lining and its contents upon surgical exposure and finally by histopathological examination. Verification of this very rare association between GGS and DCs may enrich the literature, and guide physicians and dentists to an accurate and early diagnosis of GGS.

## Consent for publication

A written consent was obtained from the patient to publish this case report.

## Ethical approval

Ethical approval was deemed unnecessary by our institutional ethical committee, as the paper is reporting a single case that emerged during normal practice.

## Funding

No funding was received to assist with the preparation of this manuscript.

## CRediT authorship contribution statement

Mohammad H Al-Shayyab: supervision, conception and design of the study, final approval of the final draft. Ra'ed Hisham Aldweik: acquisition of data, investigations necessary for the report, drafting the article. Mohammad Alzyoud and Aya Qteish: acquisition of data, investigations, in particular histopathological examination and interpretation necessary for the report. All authors read and approved the final manuscript.

## Guarantor

Mohammad H Al-Shayyab.

## Research registration number

1. Name of the registry: not applicable
2. Unique identifying number or registration ID: not applicable
3. Hyperlink to your specific registration (must be publicly accessible and will be checked): not applicable

## Declaration of competing interest

The authors declare that they have no known competing financial interests or personal relationships that could have appeared to influence the work reported in this paper.
